# Editorial: Featural Relations in the Brain: Theoretical and Experimental Perspectives on Grammatical Agreement

**DOI:** 10.3389/fpsyg.2021.754430

**Published:** 2021-10-08

**Authors:** Simona Mancini, Sendy Caffarra, Andrew Nevins

**Affiliations:** ^1^Basque Center on Cognition, Brain and Language, San Sebastian, Spain; ^2^Division of Developmental-Behavioral Pediatrics, Stanford University School of Medicine, Stanford, CA, United States; ^3^Stanford University Graduate School of Education, Stanford, CA, United States; ^4^Department of Biomedical, Metabolic and Neural Sciences, University of Modena and Reggio Emilia, Modena, Italy; ^5^Division of Psychology and Language Sciences, University College London, London, United Kingdom

**Keywords:** syntax, agreement, sentence comprehension, sentence production, morphosyntactic features

Theoretical linguistics has provided an articulated system of structural representations and computations on which the establishment of agreement relations hinges, while psycholinguistics and neurolinguistics aim at unveiling the algorithms that underlie the use of these computations and their behavioral and neurophysiological bases. The goal of this special issue is to describe the state of the art in the theoretical and experimental study of agreement. Its 15 articles open a unique and privileged window onto a wide range of languages (from English and German to Romance languages like Italian, French, and Spanish, but also to less well-studied languages within psycholinguistics and neurolinguistics such as Georgian, Korean, Standard Modern Arabic, and South Slavic languages), through the lens of distinct features (person, gender, number, and tense), drawing evidence from a variety of experimental paradigms (e.g., offline elicitation tasks, self-paced reading, eye tracking, and event-related potentials) and diverse theoretically-grounded approaches.

Three main take-home messages emerge from the articles collected in this special issues. First, agreement does not a constitute a monolithic phenomenon: person, number, gender, and tense features have inherent structural and interpretive differences, which produce common but also feature-specific reflexes in comprehension and production. Second, the mechanisms that guide the parser in retrieving and encoding features during the building of an agreement relation obey distinct principles, depending on whether features are structurally accessible or not. Finally, agreement is not wholly circumscribed within syntax: its comprehension and production trigger the integration of information from distinct linguistic and non-linguistic domains. Let us see these points in more detail.

## Distinct Features, Distinct Mechanisms? Against Features as Uniform Constructs

A landmark of numerous theoretical analyses within the generative framework is the idea that agreement features cannot be treated as a “bundle” under the same T head (Chomsky, 2014). The intrinsically different syntactic and interpretive properties that characterize e.g., person, number, tense, and gender agreement make it plausible to hypothesize the independent representation of such features (Shlonsky, 2010; Sigurdsson, 2010; Rizzi and Cinque, 2016, among others). Hartmann and Heycock contribute to this research line by showing how person and number features can be structurally differentiated in several Germanic languages, such as Dutch, Faroese, German, and Icelandic.

A prolific strand of experimental research has been also devoted to investigating whether features' distinct representational properties have a processing reflex, of which the person-number dissociation hypothesis has been one of the main testing grounds (Nevins et al., 2007; Silva-Pereyra and Carreiras, 2007; Mancini et al., 2011, 2017; Zawiszewski et al., 2016; Biondo et al., 2018 to name a few). Existing findings attributed qualitative differences in their processing to the different interpretive properties that characterize the two types of agreement: the link to discourse participant roles that is necessary to interpret 1st, 2nd, or 3rd person (a speaker, an addressee or a non-active participant), but not the singularity or the plurality of the individuals involved in the speech event. Interestingly, an alternative explanation is proposed here by den Dikken that centers on the distinct checking mechanisms in which the two features engage: both spec-head and Agree for number, while only spec-head for person.

While experimental studies overall agree that the response to agreement violations is stronger when person, rather than number is involved, they diverge on whether qualitative (Mancini et al., 2011, 2017; Biondo et al., 2018) or quantitative (Zawiszewski et al., 2016) differences emerge between these two features. Ackema and Neeleman's analysis proposes that the type of agreement controller and the distinct feature sets that pronouns and regular noun phrases (NPs) carry can reconcile these apparently contradicting results.

The literature on *gender* comprehension and production also corroborates the hypothesis that agreement features cannot be treated as uniform constructs, and that their granular properties do matter for comprehension and production. In particular, Wang and Schiller show that the strength of morphophonological representations determines whether speakers access gender information through a form-related route (as happens in Romance languages) or through a lexically-based route (as for example in German and Dutch). Moreover, speakers of different linguistic profiles (i.e., monolinguals and bilinguals) are sensitive to distributional differences between masculine and feminine classes, as Beatty-Martínez and Dussias's contribution reveals.

Other fine-grained aspects of agreement controllers and targets can play a crucial role in the establishment and comprehension of relations among words. Data from Spanish (Bañón and Rothman) and Georgian (Foley and Wagers) show that factors such as the morphological markedness of the subject and the canonicity of the verb form shape the parser's expectations, and thus its sensitivity to detecting errors and initiating reanalysis processes when anomalies are encountered (see also Tucker et al.'s contribution based on data from Modern Standard Arabic for a similar finding on how the morphological markedness of the verb impacts error detection on number and gender verbal agreement).

Thus, there are multiple fine-grained distinctions that can be made when we investigate agreement mechanisms. These specific differentiations can be based on the type of grammatical features involved, their distributional properties and their morphological markedness.

## What Guides the Analysis and Interpretation of Features During Agreement Processing?

An extremely productive line of theoretical and experimental research on agreement has focused on attraction, the phenomenon whereby the production of the correct number inflection on the verb can be disrupted by the presence of an intervening plural noun phrase, as in *The key to the cabinets were rusty* (Bock and Miller, 1991). In comprehension, attraction leads to illusions of grammaticality, i.e., to the acceptance of agreement anomalous sentences (Pearlmutter et al., 1999). Several psycholinguistic accounts exist that have attempted to explain the mechanisms behind agreement attraction in comprehension and production, among which the so-called retrieval accounts. Under this theoretical framework, attraction is an error of the memory system, whereby the cues of a certain head should be retrieved but the parser can select the wrong NP if there is a partial overlap in features. Using a variety of experimental paradigms, several papers in our special issue test retrieval accounts, reporting interesting findings across typologically different languages.

Parker and An suggest that in English, attraction depends on both retrieval and encoding mechanisms and that it is sensitive to both the semantic and syntactic properties of the attractor. Interestingly, Schlueter et al. show that the attractor can be erroneously interpreted as the thematic subject and that this is orthogonal to whether attraction happens. In their study in Korean, Kwon and Sturt show that misretrieval is more likely to occur if the distractor is nominative, rather than e.g., dative-marked, suggesting that at least in languages that overtly mark case, the grammatical role of a nominal element plays a more crucial role than e.g., mere proximity to the verb.

Are all linguistic features candidate cues that guide retrieval? Are all cues given similar weight? Biondo et al., address this question in an eye tracking study in English where they test readers' sensitivity to temporal concordance between an adverb and two verbs, a structurally accessible and a structurally inaccessible verb. They show that readers were sensitive to feature match between the adverb and a linearly distant but structurally accessible verb, while the evidence about the interference of a structurally inaccessible verb is not clear.

Tucker et al. show that inherent differences between features play a role also during the processing of attraction phenomena. Indeed, in Modern Standard Arabic subject-verb agreement, gender effects are larger and surface slightly later than number attraction effects, which calls for a revision of real-time models of agreement that posit the bundling of the two features in the computation of subject-verb agreement.

The emerging picture from all these results point to a diversity of agreement mechanisms, highlighting the differential impact of the grammatical role of nominal constituents and the structural accessibility of grammatical features that are retrieved and encoded in the real time computation of agreement relations.

## Agreement Beyond Syntax

Another important question that experimental and theoretical research on agreement aim to answer is whether its mechanisms and representations are circumscribed within syntax. Based on data from Serbo-Croatian, Mitić and Arsenijević suggest that the computation of agreement relations spans beyond purely syntactic boundaries and involves the interface between syntax and phonological form, in line with accounts that place some of the computation of agreement in the post-syntactic component (Bobaljik, 2008; Arregi and Nevins, 2012).

Further evidence for the impact of extra-syntactic factors in agreement processing comes from the analysis of online and offline patterns elicited by object cleft sentences. In their study on Italian cleft sentences, Chesi and Canal manipulate whether the subject and object NPs are 3rd person or 2nd person definite NPs in various combinations, in the attempt to elucidate the role played by the properties of different NPs and different persons. By collecting acceptability judgments and accuracy data from comprehension questions, as well as online reading times from eye tracking, Chesi and Canal show that sentence processing difficulty is not wholly driven by computing the syntactic analysis. Rather, there are aspects of the interpretation and discourse factors that play a major role.

Finally, Courteau et al. attempt to cast light onto how information across visual and linguistic domains impacts processing, thus shifting the focus on how agreement between the information contained in different domains is integrated. The results of their ERP study show that participants immediately detected number mismatches between pictures and acoustically-presented, grammatically correct linguistic material. These mismatches are processed in a way that is not fundamentally different from purely linguistic, within-sentence agreement violations, thus underlining the role of contextual information in the processing of agreement dependencies.

This set of papers thus highlight potential interactions between agreement mechanisms and non-linguistic domains, such as phonology, picture-based context, and discourse.

## Conclusion

Agreement is a widespread and varied phenomenon: its pervasiveness in some languages contrasts with its near absence in others, which poses a challenge for linguists and psycholinguists that attempt to explain the mechanics of its representation and processing (Corbett, 2006). These inherent complexities notwithstanding, the 15 articles presented in this special issue clearly represent a step forward in the description of the architecture and mechanisms underlying this core linguistic function in terms of its representation and processing. The emerging picture from this collection of papers is that the mechanisms of grammatical agreement may be flexible, feature-specific, and in part non-strictly syntactic. We hope that the breadth of empirical contributions, novel methodological designs, and theoretical refinements presented herein pave the way for continued avenues of exploration of this pervasive aspect of natural language.

## Author Contributions

SM, SC, and AN equally contributed the development and running of the Research Topic, as well as to the writing of the editorial manuscript.

## Funding

This work was supported by the Basque Government through (BERC 2018-2021 program) and by the Spanish State Research Agency (BCBL Severo Ochoa excellence accreditation SEV-2015-0490). SM acknowledges funding from the Spanish Ministry of Economy, Industry and Competitiveness (RYC-2017-22015), the Spanish Ministry of Science and Innovation (PID2020-113945RB-I00), and the Basque Government (PIBA_2020_1_0024). SC acknowledges funding from the Rita Levi Montalcini grant (Italian Ministry of Research).

## Conflict of Interest

The authors declare that the research was conducted in the absence of any commercial or financial relationships that could be construed as a potential conflict of interest.

## Publisher's Note

All claims expressed in this article are solely those of the authors and do not necessarily represent those of their affiliated organizations, or those of the publisher, the editors and the reviewers. Any product that may be evaluated in this article, or claim that may be made by its manufacturer, is not guaranteed or endorsed by the publisher.

## References

[B1] Arregi K. Nevins A. (2012). Morphotactics: Basque Auxiliaries and the Structure of Spellout, Vol. 86. New YorK, NY; London: Springer Science & Business Media.

[B2] Biondo N. Vespignani F. Rizzi L. Mancini S. (2018). Widening agreement processing: a matter of time, features and distance. Lang. Cogn. Neurosci. 33, 890–911. 10.1080/23273798.2018.1446542

[B3] Bobaljik J. D. (2008). Paradigms (optimal and otherwise): a case for Scepticism. Available online at: http://www.unice.fr/scheer/egg/BLuka18/Bobalijk(2008).pdf

[B4] Bock K. Miller C. A. (1991). Broken agreement. Cogn. Psychol. 23, 45–93. 10.1016/0010-0285(91)90003-7 2001615

[B5] Chomsky N. (2014). The Minimalist Program. Cambridge, MA: MIT Press.

[B6] Corbett G. (2006). Agreement. Oxford: Oxford University Press.

[B7] Mancini S. Molinaro N. Rizzi L. Carreiras M. (2011). A person is not a number: discourse involvement in subject–verb agreement computation. Brain Res. 1410, 64–76. 10.1016/j.brainres.2011.06.055 21798519

[B8] Mancini S. Quiñones I. Molinaro N. Hernandez-Cabrera J. A. Carreiras M. (2017). Disentangling meaning in the brain: left temporal involvement in agreement processing. Cortex 86, 140–155. 10.1016/j.cortex.2016.11.008 27984788

[B9] Nevins A. Dillon B. Malhotra S. Phillips C. (2007). The role of feature-number and feature-type in processing Hindi verb agreement violations. Brain Res. 1164, 81–94. 10.1016/j.brainres.2007.05.058 17658491

[B10] Pearlmutter N. J. Garnsey S. M. Bock K. (1999). Agreement processes in sentence comprehension. J. Memory Lang. 41, 427–456. 10.1006/jmla.1999.2653

[B11] Rizzi L. Cinque G. (2016). Functional categories and syntactic theory. Annu. Rev. Linguist. 2, 139–163. 10.1146/annurev-linguistics-011415-040827

[B12] Shlonsky U. (2010). The cartographic enterprise in syntax. Lang. Linguist. Compass 4, 417–429. 10.1111/j.1749-818X.2010.00202.x

[B13] Sigurdsson H. Á. (2010). Language quarks. Iberia Int. J. Theor. Linguist. 1, 169–183. Available online at: http://www.siff.us.es/iberia/index.php/ij/article/view/6/2

[B14] Silva-Pereyra J. F. Carreiras M. (2007). An ERP study of agreement features in Spanish. Brain Res. 1185, 201–211. 10.1016/j.brainres.2007.09.029 17963736

[B15] Zawiszewski A. Santesteban M. Laka I. (2016). Phi-features reloaded: an event-related potential study on person and number agreement processing. Appl. Psycholinguist. 37, 601–626. 10.1017/S014271641500017X

